# Interferenceless coded aperture correlation holography with point spread holograms of isolated chaotic islands for 3D imaging

**DOI:** 10.1038/s41598-022-08694-z

**Published:** 2022-03-16

**Authors:** Nitin Dubey, Joseph Rosen

**Affiliations:** School of Electrical and Computer Engineering, Ben-Gurion University of the Negev, P.O. Box 653, 8410501 Beer-Sheva, Israel

**Keywords:** Optics and photonics, Imaging and sensing

## Abstract

Interferenceless coded aperture correlation holography (I-COACH) is an incoherent digital holographic technique with lateral and axial resolution similar to a regular lens-based imaging system. The properties of I-COACH are dictated by the shape of the system’s point response termed point spread hologram (PSH). As previously shown, chaotic PSHs which are continuous over some area on the image sensor enable the system to perform three-dimensional (3D) holographic imaging. We also showed that a PSH of an ensemble of sparse dots improves the system’s signal-to-noise ratio (SNR) but reduces the dimensionality of the imaging from three to two dimensions. In this study, we test the midway shape of PSH, an ensemble of sparse islands distributed over the sensor plane. A PSH of isolated chaotic islands improves the SNR of the system compared to continuous chaotic PSH without losing the capability to perform 3D imaging. Reconstructed images of this new system are compared with images of continuous PSH, dot-based PSH, and direct images of a lens-based system. Visibility, SNR, and the product of visibility with SNR are the parameters used in the study. We also demonstrate the imaging capability of a system with partial annular apertures. The reconstruction results have better SNR and visibility than lens-based imaging systems with the same annular apertures.

## Introduction

Imaging a three-dimensional (3D) scene from a single viewpoint has been a desired technological goal since the mid-twentieth century^1^. One of the technologies to achieve this goal is digital holography. In digital holography, an optical interferometer for creating a hologram is combined with a digital computer for reconstructing the image numerically^2–4^. The common ways to numerically reconstruct a 3D image from a recorded hologram are Fresnel backpropagation^5,6^, correlation techniques^7–9^, and iterative reconstruction methods^10,11^. Fresnel incoherent correlation holography (FINCH)^12^ and coded aperture correlation holography (COACH)^13^ are examples of incoherent self-interference digital holographic methods, where the image is reconstructed by the Fresnel backpropagation in FINCH and by correlation techniques in COACH. Recently, an incoherent imaging technique has been developed with the capability of 3D imaging from a single viewpoint called interferenceless coded aperture correlation holography (I-COACH)^14^. Due to the interferenceless property, the optical configuration of I-COACH is as simple as a lens-based direct imaging system. Due to the flexibility of I-COACH, it has further been used in applications like partial^15^ and synthetic^16^ aperture systems, endoscopic system^17^, resolution enhancement^18^, extending the field of view^19^, coherent imaging^20^, and imaging through scattering layer^21^.

In I-COACH^14^, incoherent light emitted from an object is modulated by a chaotic coded phase mask (CPM) and recorded by a digital camera as an object hologram (OH). This OH is cross-correlated with a library of point spread holograms (PSHs), where each PSH is a priory recorded by positioning a point source at a different axial distance from the system aperture. The cross-correlation results yield the 3D image of the original object. However, the first generation of I-COACH suffered from a relatively low signal-to-noise ratio (SNR) because the PSH had a light intensity distributed over a relatively wide detection area. Hence the signal per pixel was lower than a regular imager, in which each point is imaged to a corresponding single point. A new PSH of a sparse dot pattern was proposed^22^ to improve the inherent low SNR of I-COACH. In sparse I-COACH, light emitted from an object point, and modulated by the CPM, is concentrated into several randomly distributed dots, while the exact dot number is selected to optimize some system features. Reconstruction results with the sparse-dot PSH have higher SNR than the previous versions of I-COACH^14–16^. However, the 3D imaging capability is lost in this new generation of I-COACH^22^. This is because the sparse dots of the PSH exist in one image plane and become spread, out-of-focus, response in every other plane. Cross-correlations with these wide-spread responses yield much lower SNR in comparison to the cross-correlation with the sparse dots. Note that the PSH of sparse dots is not the only option to improve the SNR. A thin annular PSH is proposed in Ref.^23^, but since only a single annular PSH is stored in the system memory, imaging of objects located at various depths has not been demonstrated.

We propose and demonstrate in this study a midway solution between the sparse-dot and the continuous chaotic PSHs. The proposed solution is an I-COACH system with a response of sparse chaotic islands. Such a solution has midway values of SNR between the high values of the sparse dots and the low values of the continuous chaotic PSH. Regarding SNR and optical efficiency, the system performance is better than the chaotic response system, worse than the system with PSH of sparse dots, but unlike the former, it has real 3D imaging capabilities. Since this proposed method is halfway between the sparse dots and the continuous chaotic distribution, the new technique is termed sparse chaotic I-COACH (SCI-COACH).

The purpose of this study is to show that the SCI-COACH can be a reasonable compromise with 3D imaging and better SNR than a system with continuous PSH. Therefore, some of the PSH features and parameters are determined arbitrarily, leaving the optimization of these parameters for future research. Previous studies^17^ indicate that in the case of sparse dots, PSH with six dots gives good reconstruction results. Therefore, we use six identical circular islands for the entire experiments herein. Only the radius of the islands is changed between several values in a search for the optimal island size. Visibility, SNR, and the product of visibility and SNR are used as figures of merit for searching the optimal size of the islands. Five Out of six islands are arranged in the shape of a pentagon, and one island is placed at the center of the pentagon. Once the optimal size of the island is found, it is used in the entire experiments of 3D imaging and partial aperture imaging. For 3D imaging, the PSH library of the imaging system is recorded by moving a point source along various axial locations.

In our previous study^17^ we examined the possibility of using an annular aperture, leaving the central part of the aperture for other purposes rather than imaging. Because of the sparse dot pattern of the PSH, the annular I-COACH^17^ could not demonstrate 3D imaging. Therefore, we adapt the SCI-COACH concept for the annular aperture system by synthesizing the annular CPM with a sparse chaotic response. The annular apertures with various thicknesses are studied. The visibility, SNR, and the product of these two are calculated, and the optimal size of the annular aperture is further used for 3D imaging experiments.

## Experiments

**Figure 1:**
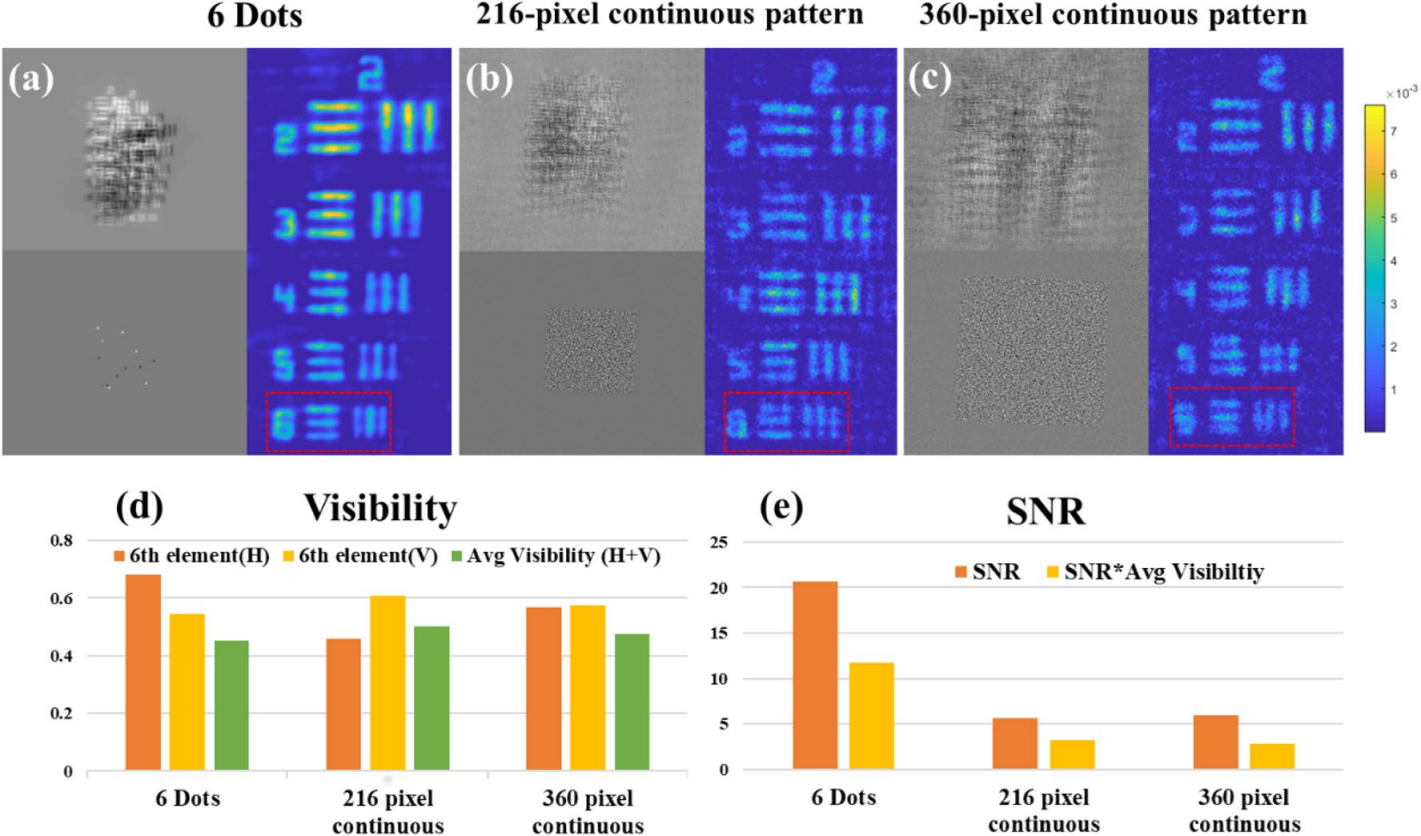
Reconstruction results of (**a**) dot pattern I-COACH system with bipolar OH and PSH, (**b**, **c**) continuous chaotic I-COACH system with constraints window of size 216 × 216 and 360 × 360 pixel along with bipolar OH and PSH, (**d**) visibility chart of 6th element, (**e**) SNR and SNR x Visibility chart.

In the first experiment, we demonstrate the differences between sparse dot PSH and continuous chaotic PSH. This comparison is the main motivation for the present imaging proposal with the midway PSH between the dot and continuous chaotic PSHs. Figure 1 shows the reconstruction results of the sparse dot response system and two cases of the continuous response system. In addition to the reconstruction, the corresponding OH (top) and PSH (bottom) are shown for each case. 12 sparse dots are used (6 negative dots and 6 positive dots) as the response system. For the continuous system, constraint windows of 216 × 216 (Fig. 1b) and 360 × 360 (Fig. 1c) pixels are chosen. By modifying the constraint window in the modified Gerchberg-Saxton algorithm (GSA)^24^, coded phase masks are created for all three cases. Although the visibility in all three cases is approximately the same, the SNR and product of visibility and SNR are highest in the sparse dot response.

Figure 1 presents two options, one with a continuous response and low SNR but with 3D imaging capability, and another is sparse dot response with high SNR but without the 3D capability. 3D imaging is one of the main advantages of holography, especially for medical applications and the entertainment industry. In order to improve the SNR, there is an option to acquire multiple images with several independent CPMs and to average over the several reconstructions^14^. However, this way of improving the SNR slows down the acquisition process. Therefore, to maintain the 3D imaging capability without sacrificing the SNR or imaging speed, we propose a compromise between continuous chaotic and sparse dot responses. The midway solution has a chaotic pattern to guarantee 3D imaging and enough sparsity to yield high enough SNR.

### Experimental setup

**Figure 2:**
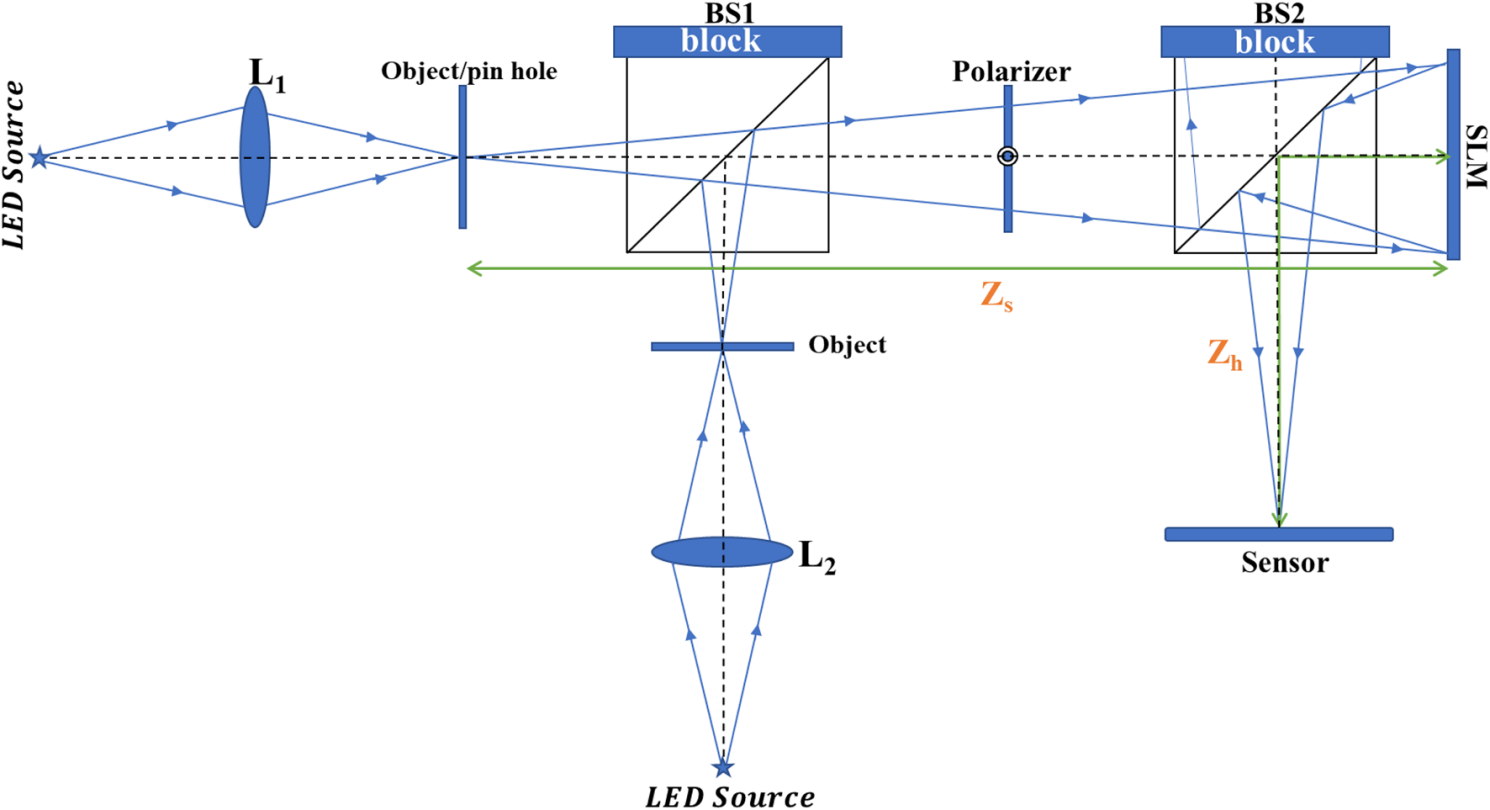
Experimental setup of SCI-COACH system.

The experimental study of SCI-COACH was carried out using the setup shown schematically in Fig. 2. A HeNe laser-illuminated a 15 μm pinhole to record the intensity response of a point object. Light diffracted from the pinhole was polarized to the active orientation of the SLM (Holoeye PLUTO, 1920 × 1080 pixels, 8 μm pixel pitch, phase-only modulation). The first beamsplitter BS1 combined the light coming from both optical channels and directed the beams toward the SLM. The phase pattern displayed on the SLM was obtained by modulo-2π phase addition of the CPM with the diffractive lens of *f* = 15 cm focal length. The beamsplitter BS2 reflected the incoming modulated light from the SLM toward a digital camera (PCO.Edge 5.5 CMOS, pixel pitch = 6.5 μm 2560 × 2160 pixel). The camera was 27 cm away from the center of the beamsplitter, and the distance between the center of the beamsplitter and SLM was 3 cm. Therefore, the distance between SLM and the camera was *z*_*h*_ = 30 cm.

For the OH, the optical setup was the same, but a LED illuminated the objects to maintain the spatial incoherence needed for the appropriate operation of SCI-COACH. A LED (Thorlabs LED 635L, 170 mW,${ \lambda }_{C}=635 \mathrm{nm},\Delta \lambda =15 \mathrm{nm}$) was mounted at a distance of 13 cm from lens L_1_ (d = 2.5 cm, f_1_ = 7 cm) and critically illuminated the object. For 3D imaging, a similar setup was constructed in the second optical channel to critically illuminate a second object with lens L_2_. A different element of the USAF resolution chart was used as an object in this study. Bipolar OH is recorded by following the same procedure of recoding the bipolar PSH. The diffractive lens was displayed on the SLM with the CPM, and the target was at a distance of z_s_ = 34 cm from the SLM.

Since the number and shape of islands are already decided, the experiment starts with optimizing the size of the chaotic islands in the intensity response. The visibility and SNR of the reconstructed images and their product are the figures of merit to finalize the optimum size of the islands. The radius of the islands is varied from *R* = 20Δ to *R* = 90Δ, where Δ is the pixel size of 8 μm In terms of microns, it started from 160 μm and finished at 720 μm with a step size of 80 μm. 6 chaotic islands of the same radius are used for each CPM to create the PSH.

### Results

The second group of the USAF chart is used as an object for the experiments. The image is reconstructed by cross-correlation between the OH and the PSH. As in the previous studies^17,22,25^, we adapt the concept of pattern recognition^26^ to the current problem by observing that the OH is an ensemble of point responses distributed over the 2D plane of the hologram. The goal of the reconstruction procedure is to convert back any point-response to a point. In other words, the cross-correlation should yield the sharpest as possible correlation peak at every position of the point-response. We followed the study in the field of pattern recognition^26^, which shows that the phase-only filter (POF) yields a sharp correlation peak with a relatively low noise level compared to the matched filter. Therefore, in this study, we use the POF correlation technique.

For the SNR, the noise was calculated by averaging the background around the reconstructed image, whereas the signal was calculated by averaging over the reconstructed image. The visibility was calculated as ν = (*I*_*max*_ − *I*_*min*_)/(*I*_*max*_ + *I*_*min*_), where *I*_*min*_ and *I*_*max*_ are the minimum and maximum intensities corresponding to the line profile, averaged over the gratings of the reconstructed object. The vertical and horizontal grating of the 6^th^ element was chosen for visibility and signal calculations.

**Figure 3:**
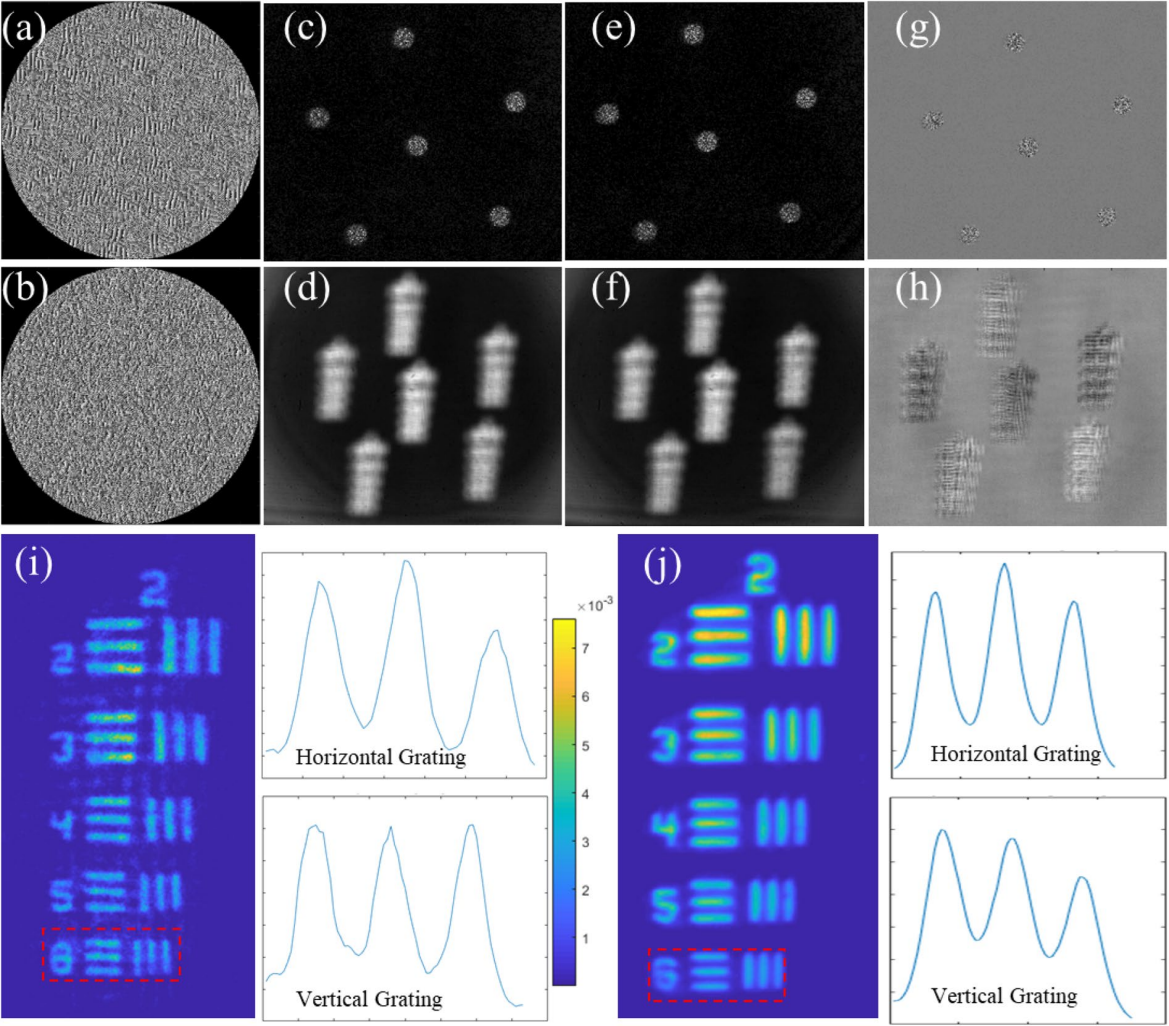
Reconstruction and imaging results of SCI-COACH and direct imaging system. (**a**, **b**) CPMs without the diffractive lens, (**c**, **e**) The two parts of the PSH, (**d**, **f**) The two parts of the object hologram, (**g**) Bipolar PSH, (**h**) Bipolar Object hologram, (**i**) SCI-COACH reconstruction result with average visibility plots of the 6th element, (**j**) Direct imaging result with average visibility plots of the 6th element.

Figure 3 shows the comparison between the SCI-COACH system with island radius *R* = 20$\Delta$ and direct imaging results. Figures 3a,b show two coded phase masks, each synthesized to yield a sparse chaotic response with radius *R* = 20$\Delta$. The PSH generated from these CPMs shown in Fig. 3c,e. Figure 3d,f show their corresponding OHs, respectively. Figure 3g,h are bipolar PSH and bipolar OH, respectively. Reconstruction result generated by cross-correlation between bipolar PSH and OH is shown in Fig. 3i along with intensity plot of the 6^th^ elements grating, whereas Fig. 3j shows the direct imaging result obtained by displaying only a diffractive lens on the SLM that satisfies the imaging condition. From the intensity profile of the grating, we conclude that SCI-COACH has the same visibility as direct imaging.

### Full aperture imaging system

**Figure 4:**
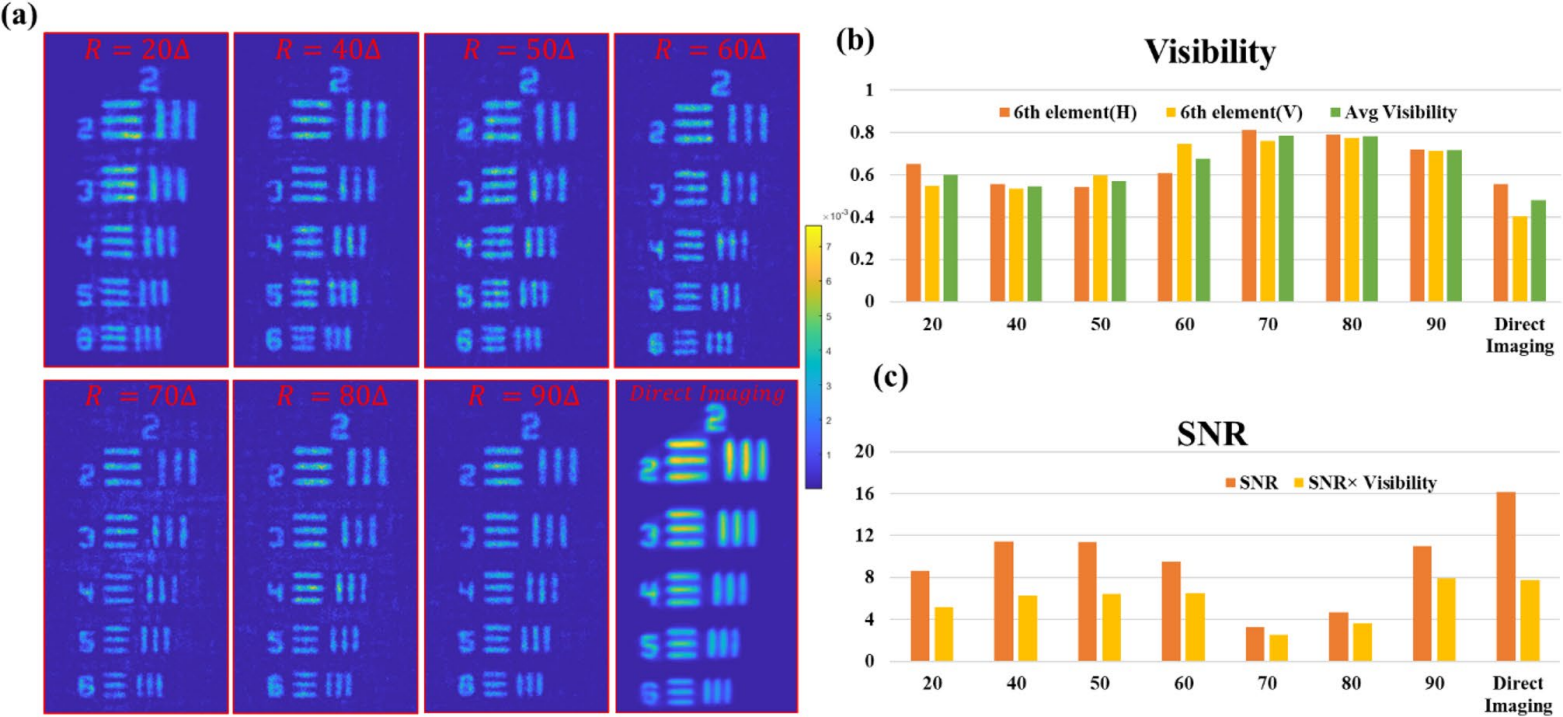
(**a**) Experimental comparison results of regular imaging and reconstruction results of the SCI-COACH system with a varying radius of the island, (**b**) Visibility chart of 6th element gratings, (**c**) SNR and SNR x Visibility chart.

An experimental study was performed to determine the optimal size of the islands for the best reconstruction quality. The reconstruction results of the SCI-COACH system are shown in Fig. 4, and the visibility, SNR, and the product of SNR × Visibility are plotted in the charts of Fig. 4b,c. The visibility of all SCI-COACH systems is better than direct imaging. Note that direct imaging has a point-to-point mapping between the object plane and the image plane, while in SCI-COACH, each point source is spread over the sensor plane in a pattern of chaotic islands. Therefore, direct imaging has a better SNR value over SCI-COACH reconstruction. However, when the product of SNR and Visibility are calculated, SCI-COACH has comparable performance as the direct imaging system, as shown in the SNR chart in Fig. 4c. Based on the charts of the SNR and SNR × visibility, SCI-COACH with *R* = 40Δ and *R* = 90Δ gives better results over other reconstruction results, and hence we choose to continue our experiment with *R* = 40Δ.

**Figure 5:**
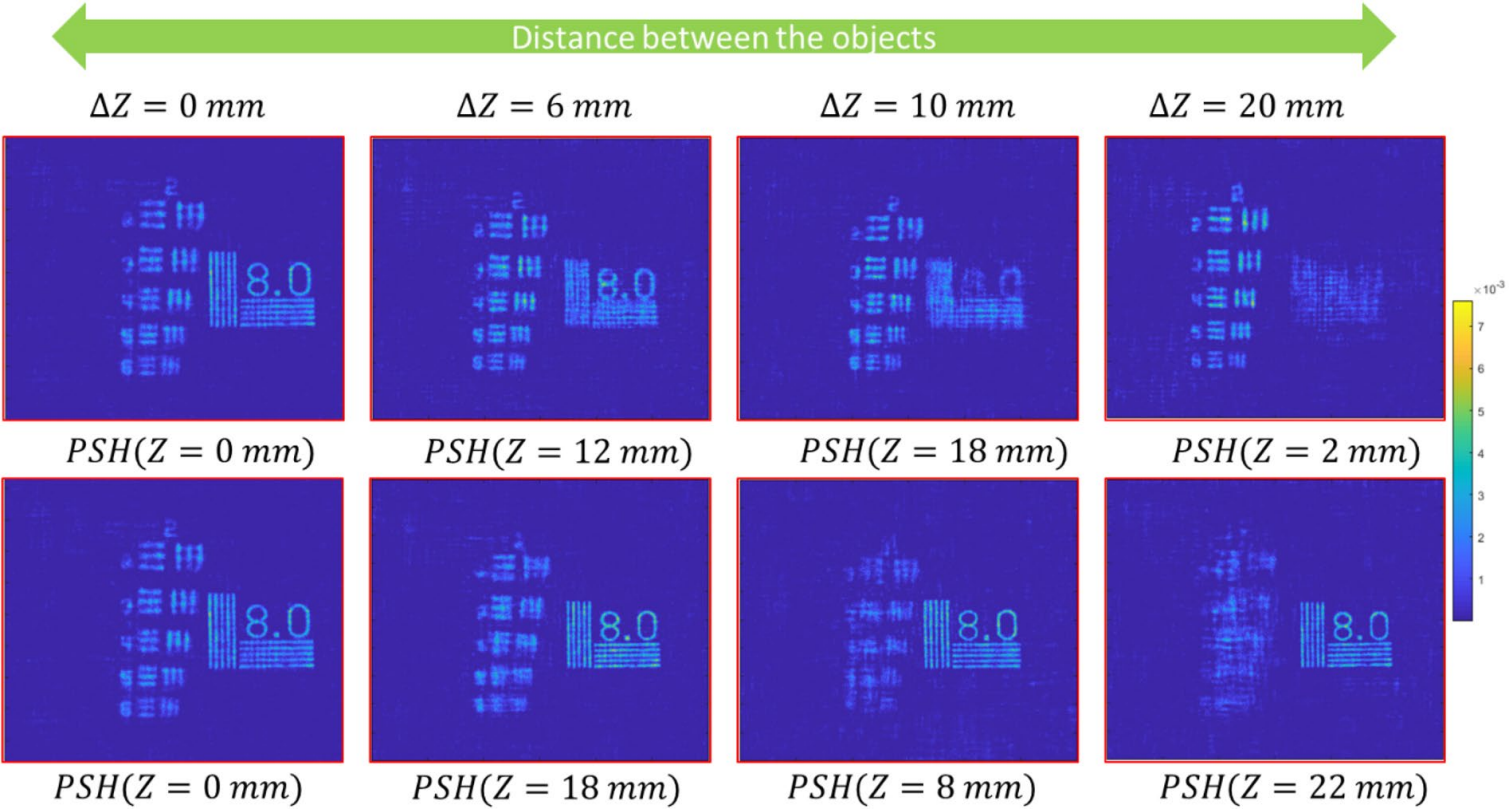
3D reconstruction results of SCI-COACH system. Two objects are placed in different axial planes with varying separation between two planes for each object hologram.

For 3D imaging, we used USAF and NBS charts, each of which at a different optical channel of the setup shown in Fig. 2 and at a different distance from BS1. The combined OHs of the targets were recorded four times for four distances between the two objects of 0, 6, 10, and 20 mm. PSHs for each axial location were a priory recorded and stored in the computer. The object reconstruction is done by cross-correlating the OHs with the PSH of the corresponding axial location. The 3D reconstruction results from OHs are shown in above Fig. 5. Next, we test the concept of SCI-COACH for the annular aperture to add the capability of 3D imaging to the previous proposed endoscopic I-COACH^17^.

### Partial aperture system

For the partial aperture system, annular coded phase masks were synthesized with the help of the modified GSA. A diffractive lens with a linear phase function replaces the central part of CPM to focus the light coming from the central part of the CPM away from the sensor. SCI-COACH of annular CPM with an annular size of 400,300,200, and 100 pixels and with island radius of *R* = 40Δ, were examined.

**Figure 6:**
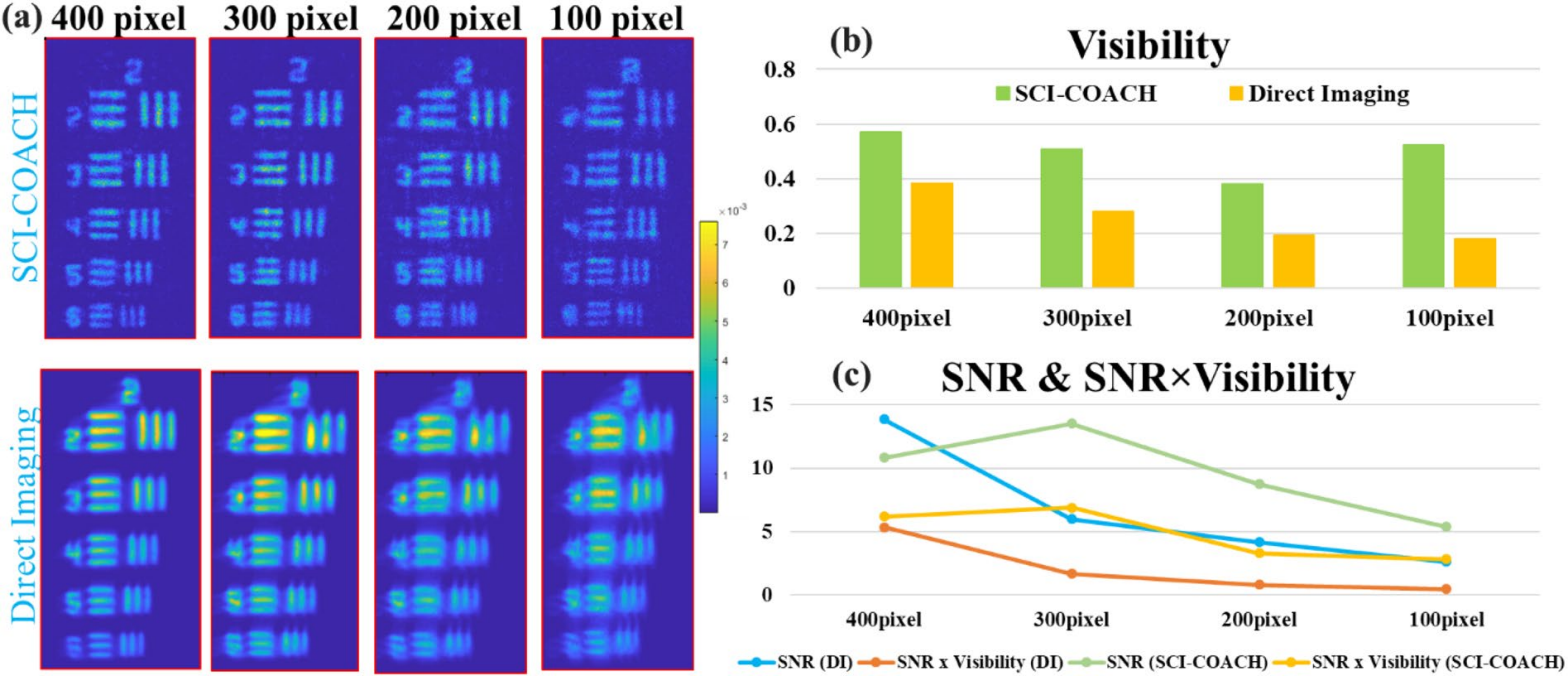
(**a**) Reconstruction and imaging results of SCI-COACH (*R* = 40 $\Delta$) and direct imaging system with annular aperture along with their (**b**) visibility, (**c**) SNR and SNR × Visibility chart.

The reconstruction results of various annular apertures are shown in Fig. 6 with the charts of visibility, SNR and SNR × Visibility. The visibility of SCI-COACH with all annular apertures is much better than the counterpart direct imaging system with the same annular aperture. The SNR chart shows that SCI-COACH is better with 300 pixels width and better SNR × visibility values for the entire width values. Therefore, the annular aperture of 300 pixels width was used for the 3D imaging test of the annular SCI-COACH.

**Figure 7:**
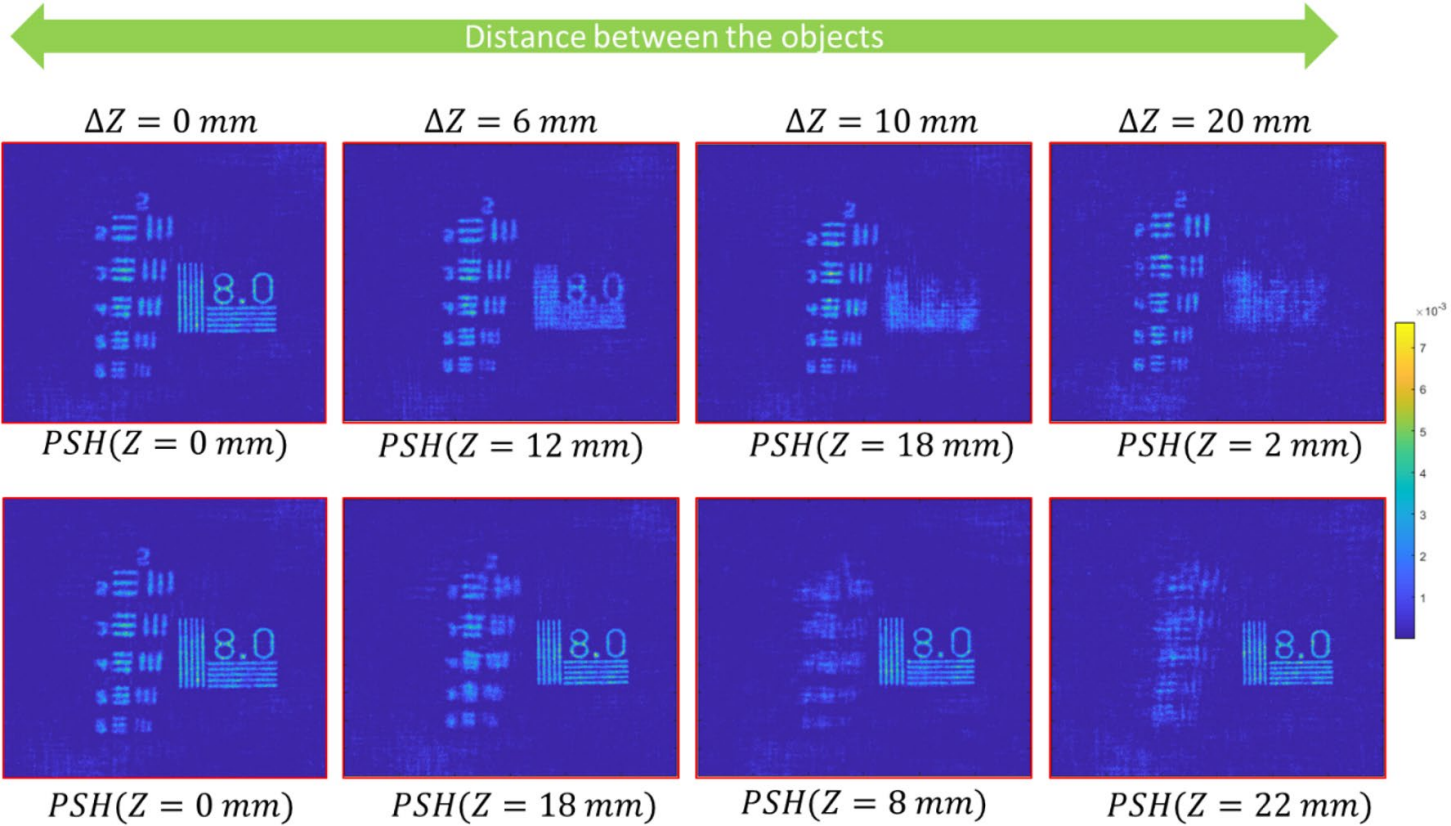
3D Reconstruction results of SCI-COACH system with an annular aperture of 300 pixels. Two objects are placed in different axial planes with varying separation between two planes.

In 3D imaging, the USAF and NBS targets were placed in the same way as the experiment of the full aperture described in Fig. 5. The PSH library was a priory created using the annular CPM. Figure 7 shows the reconstruction results from different OHs, generated by cross-correlating with the PSH of the corresponding axial location.

## Discussion and conclusion

In this study, it has been shown that by introducing sparsity in the chaotic response, the system capability to perform 3D imaging is achieved with higher SNR than the original I-COACH of the continuous chaotic response. We demonstrated that the SCI-COACH is a midway solution between the 2D I-COACH with the sparse dot response and the 3D I-COACH with the continuous chaotic response. Furthermore, we have shown that this midway solution can also be applied for the case of annular apertures. In systems like endoscopes and borescopes where space might be under constraints of additional instruments and equipment attached to the imaging system, SCI-COACH can be a solution for 3D imaging with accepted SNR values.

**Figure 8:**
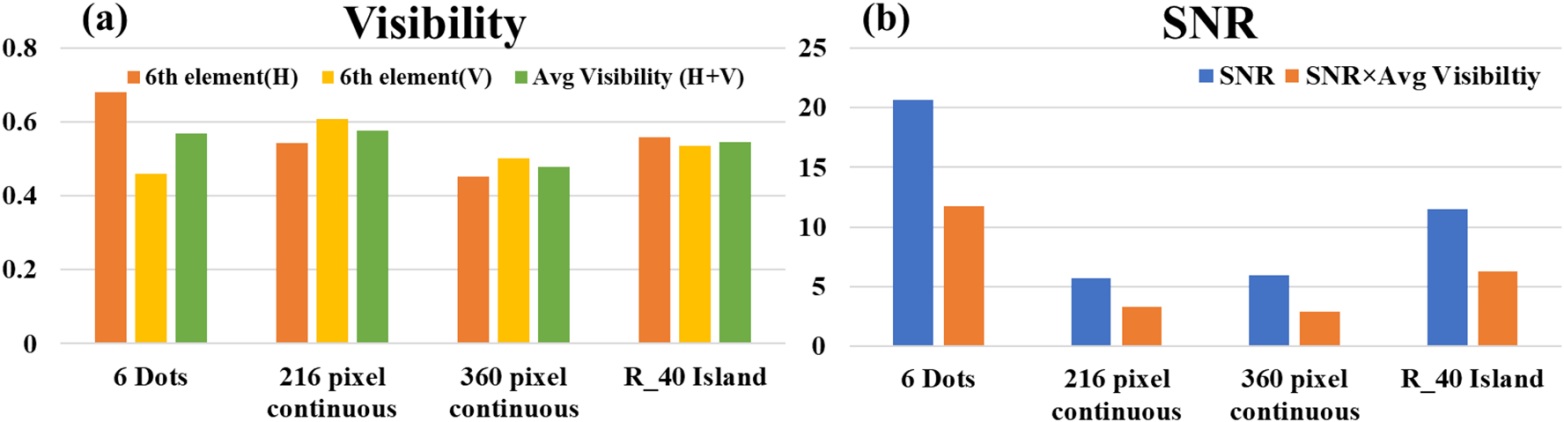
Comparative charts of (**a**) visibility and (**b**) SNR for I-COACH system’s with different system response.

The charts of Fig. 8 summarize the contribution of this study. Reconstruction results of I-COACH with PSH of 6 sparse dots, continuous chaotic with a window of 216 × 216 and 360 × 360 pixels are compared with SCI-COACH (R = 40 $\Delta$). The visibility of the various systems is shown in Fig. 8a, while SNR and SNR × visibility are plotted in Fig. 8b. Among the systems discussed in this study, the system with PSH of sparse dots has the best performance, but as mentioned above, this system is limited to 2D imaging. When we compare the 3D imaging systems, SCI-COACH has better SNR and similar visibility compared to the two options of the continuous chaotic PSH. Regarding the parameter of SNR × visibility, SCI-COACH is found better than the two other 3D options.

**Figure 9:**
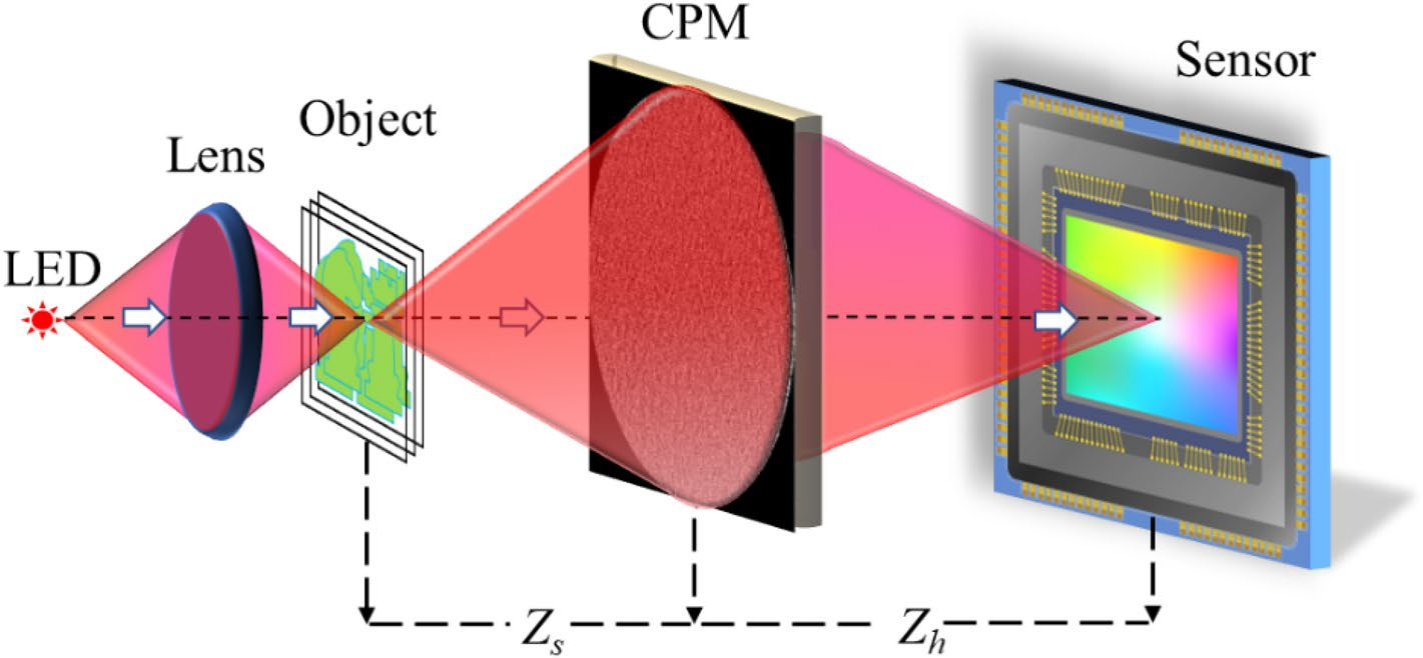
Schematic representation of the optical configuration of sparse chaotic I-COACH system.

This study proposes a solution of PSH for I-COACH systems to improve the SNR without losing the capability of 3D imaging. The main contribution of this study is the development of PSHs that produces accepted-quality SNR with the ability to perform 3D imaging. Additionally, we also demonstrate the imaging capability of I-COACH with annular apertures. Visibility, SNR, and their product are the parameters used in the study to compare images of continuous PSH, dot-based PSH, and direct imaging by a lens-based system. The central insight of the PSH design is a pattern of a few isolated, chaotic islands. The pattern of the isolated, chaotic islands is achieved by the use of GSA, a well-known algorithm proposed in the past as a solution for the phase retrieval problem. Although the limitations and drawbacks of GSA for the phase retrieval problem were extensively analyzed recently^27^, the current problem is different from the phase retrieval problem. The problem of finding an arbitrary phase mask that induces isolated, chaotic islands on the camera plane is different from retrieving a specific phase distribution that yields some measured intensity distribution in the far-field. Therefore, the limitations pointed out in Ref. ^27^ are not valid for the present case. Note that a phase mask design increases the quality of the reconstructed images. Therefore, the design of the phase masks is executed by the GSA, which is the best method we know today to design phase masks for the present task. Different methods of phase mask design proposed for the quantitative reconstruction of complex-valued objects^28^ are not applicable here because 1. The present imaging task is different than the quantitative reconstruction of complex-valued 2D objects. 2. The setup and the operation of the phase mask in Ref. 28 are completely different than the setup of Fig. 9 and the operation as an aperture of an incoherent imaging system.

## Methodology

The scheme of I-COACH with sparse chaotic point response is shown in Fig. 9. The SLM modulates light emitted from a point source, which comprises CPM with a diffractive lens of focal length *f*. The light modulated by CPM is projected on the sensor plane by the diffractive lens to satisfy the Fourier-transform relation between the CPM and sensor planes. By removing CPM from the SLM, the system acts as a direct imaging system. The image from the direct imaging system is further used as a reference compared to the proposed SCI-COACH, the chaotic continuous I-COACH, and the dot-based I-COACH.

The spatially incoherent, quasi-monochromatic source of light is used to critically illuminate the object. Hence, the system is treated as a spatially incoherent imaging system with linear space-invariant relations between the intensity patterns on the camera and on the object plane. The following mathematical analysis is based on the optical configuration of Fig. 9. Considering a point object located at $\left( {\bar{{r_{s} }} , - z_{s} } \right) = \left( {x_{s} ,y_{s} , - z_{s} } \right)$ with an amplitude $\sqrt {I_{s} }$, the complex amplitude just before SLM is given as,

1 $$I_{k} \left( {\bar{r}_{{}} ;\bar{r}_{s} ,z_{s} } \right) = \sqrt {I_{s} } C_{0} L\left( {\frac{{\bar{r}_{s} }}{{z_{s} }}} \right)Q\left( {\frac{1}{{z_{s} }}} \right),$$

where *L* and *Q* represent linear and quadratic phase functions, given by $L\left( {\bar{s}/z} \right) = \exp \left[ {i2\pi \left( {\lambda z} \right)^{ - 1} \left( {s_{x} x + s_{y} y} \right)} \right]$ and $Q\left( a \right) = \exp \left[ {i\pi a\lambda^{ - 1} \left( {x^{2} + y^{2} } \right)} \right],$ respectively. *λ* is the wavelength, and *C*_0_ is a complex constant. The complex amplitude modulated by CPM is given by,

2 $$I_{k} \left( {\bar{r}_{{}} ;\bar{r}_{s} ,z_{s} } \right) = \sqrt {I_{s} } C_{0} L\left( {\frac{{\bar{r}_{s} }}{{z_{s} }}} \right)Q\left( {\frac{1}{{z_{s} }}} \right)Q\left( { - \frac{1}{{f_{0} }}} \right)\exp \left[ {i\Theta_{k} \left( r \right)} \right],$$

where $\Theta_{k} \left( r \right)$ is the *k*th pseudorandom phase of the CPM calculated using the modified GSA. The complex amplitude at the image sensor is given as 2D convolution between Eq. (2) and quadratic phase function $Q\left( {{1 \mathord{\left/ {\vphantom {1 {z_{h} }}} \right. \kern-\nulldelimiterspace} {z_{h} }}} \right)$ for distance *z*_*h*_. Therefore, the intensity pattern on the image sensor is given by,

3 $$I_{k} \left( {\bar{r}_{0} ;\bar{r}_{s} ,z_{s} } \right) = \left| {\sqrt {I_{s} } C_{0} L\left( {\frac{{\bar{r}_{s} }}{{z_{s} }}} \right)Q\left( {\frac{1}{{z_{s} }}} \right)Q\left( { - \frac{1}{{f_{0} }}} \right)\exp \left[ {i\Theta_{k} \left( r \right)} \right]*Q\left( {\frac{1}{{z_{h} }}} \right)} \right|^{2} ,$$

where $*$ is a sign of 2D convolution and $\bar{r}_{0} = \left( {u,v} \right)$ is the transverse location vector on the sensor plane. The light diffracted from the CPM is Fourier transformed by the diffractive lens of focal length $f_{0}$, on the image sensor located at a distance of *z*_*h*_^22^.

4 $$I_{k} \left( {\bar{r}_{0} ;\bar{r}_{s} ,z_{s} } \right) = \left| {\nu \left[ {\frac{1}{{\lambda z_{h} }}} \right]\mathfrak{F}\left\{ {\sqrt {I_{s} } C_{0} L\left( {\frac{{\bar{r}_{s} }}{{z_{s} }}} \right)Q\left( \xi \right)\exp \left( {i\Theta_{k} \left( r \right)} \right)} \right\}} \right|^{2} = I_{k} \left( {\bar{r}_{0} - \frac{{z_{h} }}{{z_{s} }}\bar{r}_{s} ;0,z_{s} } \right)$$

where $\xi = {{\left( {f_{0} z_{s} + f_{0} z_{h} - z_{s} z_{h} } \right)} \mathord{\left/ {\vphantom {{\left( {f_{0} z_{s} + f_{0} z_{h} - z_{s} z_{h} } \right)} {f_{0} z_{s} z_{h} }}} \right. \kern-\nulldelimiterspace} {f_{0} z_{s} z_{h} }},$ $\mathfrak{F}$ is the 2D Fourier transform operator, and $\nu$ is the scaling operator defined by $\nu \left[ a \right]f\left( x \right) = f\left( {ax} \right)$. The object intensity response on the sensor plane is a shifted version [by $\left( {z_{h} /z_{s} } \right)\bar{r}_{s}$] of the intensity response of a point object at $\bar{r}_{s} = \left( {0,0} \right)$.

A 2D object at a distance *z*_*s*_ from the SLM can be considered as a collection of *N* uncorrelated point objects given as $o\left( {\overline{r}_{s} } \right) = \sum\nolimits_{j}^{N} {a_{j} \delta \left( {\overline{r}_{s} - \overline{r}_{j} } \right)}$, where $a_{j}$ is the intensity of the *j*th object point at $\overline{r}_{j}$. The object is illuminated by an incoherent quasi-monochromatic light, and therefore there is no interference between the individual point responses due to the spatial incoherence of the object light. The overall intensity distribution on the sensor plane is a sum of the point responses given by $I_{OBJ} \left( {\overline{r}_{0} ;z_{s} } \right) = \sum\nolimits_{j} {a_{j} } I_{k} \left( {\overline{r}_{0} - \left( {z_{h} /z_{s} } \right)\overline{r}_{j} ;0,z_{s} } \right)$. $I_{OBJ} \left( {\overline{r}_{0} ;z_{s} } \right)$ and $I_{k} \left( {\overline{r}_{0} ;z_{s} } \right)$ are both positive real functions with dominant bias terms. Therefore, images reconstructed by a cross-correlation between $I_{k} \left( {\overline{r}_{0} ;z_{s} } \right)$ and $I_{OBJ} \left( {\overline{r}_{0} ;z_{s} } \right)$ yield an undesired background distribution. Since the average intensity of any response generated with any CPM is approximately the same, the difference distribution between any two intensity responses almost lacks bias. Therefore, in order to minimize the background distribution, both $H_{PSH} \left( {\overline{r}_{0} ;z_{s} } \right)$ and $H_{OBJ} \left( {\overline{r}_{0} ;z_{s} } \right)$ become bipolar by capturing two shots with different CPMs, as follows,

5 $$\begin{aligned} H_{PSH} \left( {\overline{r}_{0} ;z_{s} } \right) & = I_{1} \left( {\overline{r}_{0} ;z_{s} } \right) - I_{2} \left( {\overline{r}_{0} ;z_{s} } \right) \\ H_{OBJ} \left( {\overline{r}_{0} ;z_{s} } \right) & = I_{OBJ,1} \left( {\overline{r}_{0} ;z_{s} } \right) - I_{OBJ,2} \left( {\overline{r}_{0} ;z_{s} } \right) \\ & = \sum\limits_{j} {a_{j} } I_{1} \left( {\overline{r}_{0} - \frac{{z_{h} }}{{z_{s} }}\overline{r}_{j} ;0,z_{s} } \right) - \sum\limits_{j} {a_{j} } I_{2} \left( {\overline{r}_{0} - \frac{{z_{h} }}{{z_{s} }}\overline{r}_{j} ;0,z_{s} } \right) \\ & = \sum\limits_{j} {a_{j} } H_{PSH} \left( {\overline{r}_{0} - \frac{{z_{h} }}{{z_{s} }}\overline{r}_{j} ;0,z_{s} } \right) \\ \end{aligned}$$

Therefore, two intensity response patterns are recorded for both the object and the point source using two different CPMs synthesized with different initial random phases.

The image is reconstructed by cross-correlating between $H_{PSH} \left( {\overline{r}_{0} ;z_{s} } \right)$ and $H_{OBJ} \left( {\overline{r}_{0} ;z_{s} } \right)$ by POF as follows,

6 $$\begin{aligned} R(\bar{r}_{t} ) & = H_{{OBJ}} \left( {\bar{r}_{0} ;z_{s} } \right) \otimes H_{{PSH}} ^{\prime } \left( {\bar{r}_{0} - \bar{r}_{t} ;z_{s} } \right) = \mathcal{F}^{{ - 1}} \left\{ {\mathcal{F}\left\{ {H_{{OBJ}} } \right\}\mathcal{F}^{*} \left\{ {H_{{PSH}} ^{\prime } } \right\}} \right\} \\ & = \mathcal{F}^{{ - 1}} \left\{ {\mathcal{F}\left\{ {\sum\limits_{j} {a_{j} } H_{{PSH}} \left( {\bar{r}_{0} - \frac{{z_{h} }}{{z_{s} }}\bar{r}_{j} ;z_{s} } \right)} \right\}\mathcal{F}^{*} \left\{ {H_{{PSH}} ^{\prime } } \right\}} \right\} \\ & = \mathcal{F}^{{ - 1}} \left\{ {\sum\limits_{j} {a_{j} } \mathcal{F}\left\{ {H_{{PSH}} \left( {\bar{r}_{0} - \frac{{z_{h} }}{{z_{s} }}\bar{r}_{j} ;z_{s} } \right)} \right\}\exp \left[ { - i\arg \left( {\mathcal{F}\left\{ {H_{{PSH}} } \right\}} \right)} \right]} \right\} \\ & = \mathcal{F}^{{ - 1}} \left\{ {\sum\limits_{j} {a_{j} } \left| {\mathcal{F}\left\{ {H_{{PSH}} \left( {\bar{r}_{0} ;z_{s} } \right)} \right\}} \right|\exp \left[ { - i\frac{{z_{h} }}{{z_{s} }}\bar{r}_{j} \cdot \bar{\rho }} \right]} \right\} \\ & = \sum\limits_{j} {a_{j} } \Lambda \left( {\bar{r}_{t} - \frac{{z_{h} }}{{z_{s} }}\bar{r}_{s} ;z_{s} } \right) \approx o\left( {\frac{{\bar{r}}}{{M_{T} }}} \right), \\ \end{aligned}$$

where Λ is a *δ*-like function ~ 1 at (0,0) and ~ 0 elsewhere, $\bar{\rho }$ is the position vector of the spatial frequency plane, and ${M}_{T}={z}_{h}/{z}_{s} .$ It must be noted that the image is reconstructed using a cross-correlation. Therefore, the transverse resolution is dictated by the transverse correlation length, determined by the width and length of the smallest spot that can be recorded on the sensor plane by the SLM with an active area of a *D* diameter (assuming that the active area is the smallest aperture in the system). Therefore, the transverse and axial resolutions are approximately 1.22*λz*_*s*_/*D* and 8*λ*(*z*_*s*_/*D*)^2^, respectively, matching with the resolution values of the regular incoherent imaging system with a similar numerical aperture (NA).

### Synthesis of the phase mask

**Figure 10:**
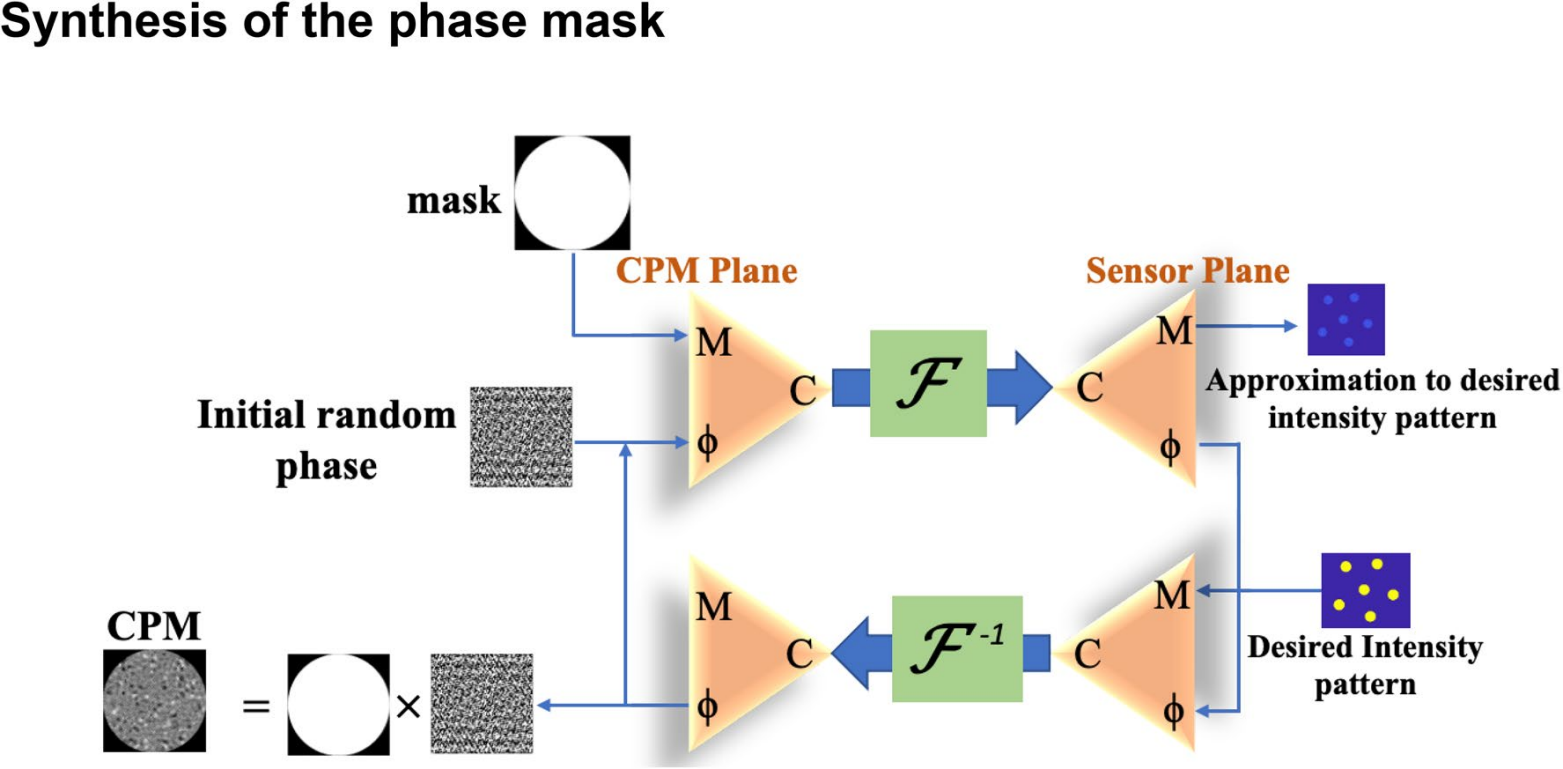
Schematic of modified GSA used for synthesizing the CPM.

The CPMs are synthesized in the computer using a modified version of the GSA shown schematically in Fig. 10. Two parameters dictate the nature of the CPMs; the first is the size of each island, and the second is the number of islands. In the case of the sparse dot pattern, it was found that 6 dots give the optimal reconstruction results in terms of SNR and visibility, and hence for the sparse chaotic island, 6 islands were chosen. The arrangement of the islands is arbitrary such that five islands are placed in a pentagon shape, and one island is in the center. By changing the size of the islands, different CPMs are tested, and the optimal CPM is used for the experimental study.

**Figure 11:**
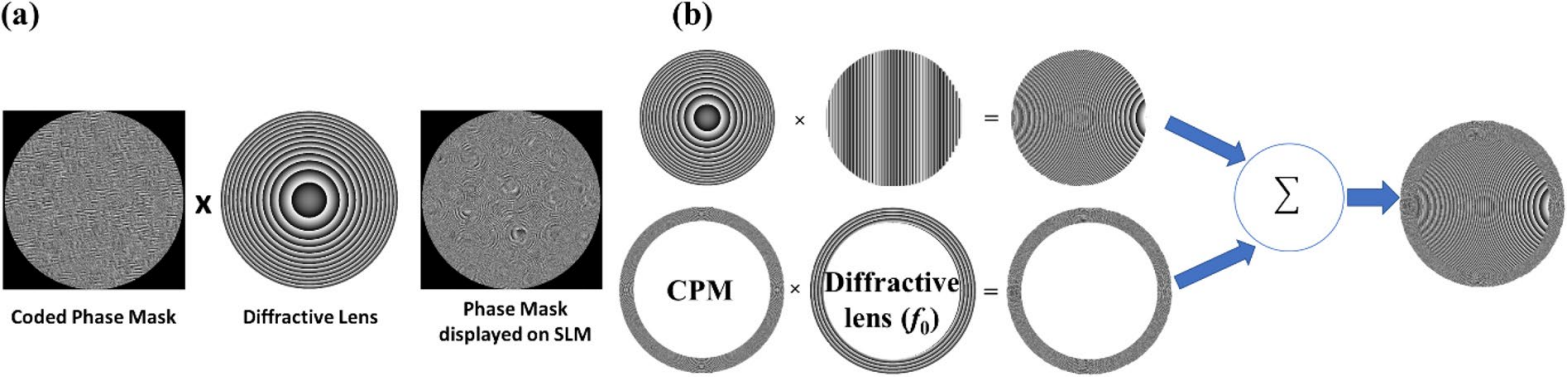
(**a**) Construction of full aperture CPM with diffractive lens and (**b**) of the annular CPM with DOE.

In the GSA of Fig. 10, an initial random phase mask is Fourier transformed from the CPM plane to the sensor plane. The magnitude distribution is replaced with the chosen pattern of chaotic islands on the sensor plane, where the phase distribution remains unchanged. The resulting complex amplitude is inversely Fourier transformed to the CPM plane, and the magnitude distribution is replaced with the uniform magnitude. This iterative process continues till the generated intensity profile on the sensor converges to satisfy the constraints. We follow the same procedure for the annular aperture, but in this case, the aperture starts with a ring-shaped phase mask instead of the uniform disk. The generated CPM is displayed on the SLM with a diffractive lens to satisfy the Fourier relation between the CPM and sensor planes. In the case of annular aperture, to focus unwanted light away from the sensor, we displayed a diffractive optical element (DOE) containing a quadratic phase function with a linear phase function. This DOE is displayed in the internal area of the SLM surrounded by the annular CPM. Therefore, only the light passing through the annular CPM arrives at the sensor. The synthesis of the complete phase mask displayed on the SLM for the full aperture system and for the partial aperture system is shown schematically in Fig. 11.
